# Hereditary Leiomyomatosis and Renal Cell Cancer Syndrome in Spain: Clinical and Genetic Characterization

**DOI:** 10.3390/cancers12113277

**Published:** 2020-11-05

**Authors:** A. Beatriz Sánchez-Heras, Adela Castillejo, Juan D. García-Díaz, Mercedes Robledo, Alexandre Teulé, Rosario Sánchez, Ángel Zúñiga, Enrique Lastra, Mercedes Durán, Gemma Llort, Carmen Yagüe, Teresa Ramon y Cajal, Consol López San Martin, Adrià López-Fernández, Judith Balmaña, Luis Robles, José M. Mesa-Latorre, Isabel Chirivella, María Fonfria, Raquel Perea Ibañez, M. Isabel Castillejo, Inés Escandell, Luis Gomez, Pere Berbel, Jose Luis Soto

**Affiliations:** 1 Cancer Genetic Counselling Unit, Medical Oncology Department, Hospital General Universitario de Elche, 03203 Elche, Spain; perea_mar@gva.es; 2 Molecular Genetics Unit, Hospital General Universitario de Elche, 03203 Elche, Spain; castillejo_ade@gva.es (A.C.); castillejo_isa@gva.es (M.I.C.); soto_jos@gva.es (J.L.S.); 3 Clinical Genetics Unit, Department of Internal Medicine, University Hospital Príncipe de Asturias, 28805 Alcalá de Henares, Spain; juandedios.garcia@salud.madrid.org (J.D.G.-D.); jmesa@salud.madrid.org (J.M.M.-L.); 4 Hereditary Endocrine Cancer Group, Spanish National Cancer Research Center (CNIO), 28029 Madrid, Spain; mrobledo@cnio.es; 5 Centro de Investigación Biomédica en Red de Enfermedades Raras, CIBERER, 28029 Madrid, Spain; 6 Hereditary Cancer Program, Catalan Institute of Oncology, Instituto de Investigación Biomédica de Bellvitge, 08908 Hospitalet de Llobregat, Spain; ateule@iconcologia.net; 7 Unidad Multidisciplinar de Enfermedades de Baja Prevalencia, Instituto de Investigación Sanitaria y Biomédica de Alicante (ISABIAL), Hospital General Universitario de Alicante, 03015 Alicante, Spain; sanchez_rosmar@gva.es; 8 Unit of Genetics, Hospital Universitario Politécnico La Fe, 46026 Valencia, Spain; zunyiga_ang@gva.es; 9 Unidad de Consejo Genético en Cáncer Hereditario, Servicio de Oncología Médica, Hospital Universitario de Burgos, 09006 Burgos, Spain; elastra@saludcastillayleon.es; 10 Genética del Cáncer, Instituto de Biología y Genética Molecular (IBGM-UVA-CSIC), 47003 Valladolid, Spain; merche@ibgm.uva.es; 11 Hereditary Cancer Unit, Medical Oncology Department, Corporació Sanitaria Universitària Parc Taulí, 08208 Sabadell, Spain; gllort@tauli.cat; 12 Hereditary Cancer Unit, Medical Oncology Department, Consorci Sanitari de Terrassa, 08191 Rubi, Spain; cyague@cst.cat; 13 Medical Oncology Department, Hospital Santa Creu i Sant Pau, 08091 Barcelona, Spain; tramon@santpau.cat (T.R.y.C.); clopezsa@santpau.cat (C.L.S.M.); 14 Hereditary Cancer Genetics Group, Medical Oncology Department (VHIO), Hospital Universitario Vall d’Hebron, 08035 Barcelona, Spain; adlopez@vhio.net (A.L.-F.); jbalmana@vhio.net (J.B.); 15 Unidad de Cáncer Familiar, Servicio de Oncología Médica, Hospital Universitario 12 de Octubre, 28041 Madrid, Spain; luis.robles@salud.madrid.org; 16 Department of Medical Oncology, INCLIVA Biomedical Research Institute, University of Valencia, 46010 Valencia, Spain; chirivella_isa@gva.es; 17 Cancer Genetic Counselling Unit, Medical Oncology Department, Consorcio Hospitalario Provincial de Castellón, 12002 Castellón, Spain; mfonfria@uji.es; 18 Servicio de Dermatología, Hospital General Universitario de Elda, 03600 Elda, Spain; escandell_ine@gva.es; 19 Urology Department, Hospital Universitario Sant Joan de Alicante, 03550 Sant Joan de Alicante, Spain; l.gomez@umh.es; 20 Departamento de Histología y Anatomía, Facultad de Medicina, Universidad Miguel Hernández, 03550 Sant Joan de Alicante, Spain; pere.berbel@umh.es

**Keywords:** leiomyomas, hereditary leiomyomatosis, *FH* gene, missense pathogenic variants, renal cell cancer

## Abstract

**Simple Summary:**

Hereditary leiomyomatosis and renal cell cancer (HLRCC) syndrome is a very rare hereditary disorder characterized by cutaneous leiomyomas (CLMs), uterine leiomyomas (ULMs), renal cysts (RCys) and renal cell cancer (RCC), with no data on its prevalence worldwide. No genotype-phenotype associations have been described. The aim of our study was to describe the genotypic and phenotypic features of the largest series of patients with HLRCC from Spain reported to date. Of 27 *FH* germline pathogenic variants, 12 were not previously reported in databases. Patients with missense pathogenic variants showed higher frequencies of CLMs, ULMs and RCys, than those with loss-of-function variants. The frequency of RCCs (10.9%) was lower than those reported in the previously published series.

**Abstract:**

Hereditary leiomyomatosis and renal cell cancer syndrome (HLRCC) is a very rare hereditary disorder characterized by cutaneous leiomyomas (CLMs), uterine leiomyomas (ULMs), renal cysts (RCys) and renal cell cancers (RCCs). We aimed to describe the genetics, clinical features and potential genotype-phenotype associations in the largest cohort of fumarate hydratase enzyme mutation carriers known from Spain using a multicentre, retrospective study of individuals with a genetic or clinical diagnosis of HLRCC. We collected clinical information from medical records, analysed genetic variants and looked for genotype-phenotype associations. Analyses were performed using R 3.6.0. software. We included 197 individuals: 74 index cases and 123 relatives. CLMs were diagnosed in 65% of patients, ULMs in 90% of women, RCys in 37% and RCC in 10.9%. Twenty-seven different pathogenic variants were detected, 12 (44%) of them not reported previously. Patients with missense pathogenic variants showed higher frequencies of CLMs, ULMs and RCys, than those with loss-of-function variants (*p* = 0.0380, *p* = 0.0015 and *p* = 0.024, respectively). This is the first report of patients with HLRCC from Spain. The frequency of RCCs was lower than those reported in the previously published series. Individuals with missense pathogenic variants had higher frequencies of CLMs, ULMs and RCys.

## 1. Introduction

More than 200 hereditary cancer susceptibility syndromes associated with specific gene mutations are known: some of these are very rare. Genetic counselling and testing allow individuals to know their risks, make choices for cancer screening, prevention and—in some forms of cancers—targeted treatment therapies. Moreover, the study of hereditary tumours has improved understanding of the molecular basis of tumorigenesis and in developing therapeutic target agents, especially for renal cancers.

In 1973, Reed et al. [1] described two families whose members presented with cutaneous leiomyomas (CLMs) and uterine leiomyomas (ULMs) with an autosomal dominant pattern of inheritance, and a 20-year-old patient with renal cell cancer (RCC). First, it was named multiple cutaneous and uterine leiomyomatosis or Reed syndrome. In 2001, Launonen et al. [2] proposed the term hereditary leiomyomatosis and renal cell cancer (HLRCC; OMIM #150800). In 2002, Tomlinson et al. [3] proved that germline heterozygous pathogenic variants in the gene encoding fumarate hydratase (*FH*) cause this syndrome, adding to the group of known hereditary renal cancer genes such as *VHL*, *MET*, *TSC1*, *TSC2*, *FLCN* and *SDH*.

Pathogenic variants in *FH* generate inactivated fumarase or fumarate hydratase enzyme (FH) and lead to failure of the tricarboxylic acid cycle (TCA), essential in cellular respiration to harvest or release energy. Heterozygous carriers have an increased risk of CLMs and ULMs that rarely become leiomyosarcomas, renal cysts (RCys) and RCCs [2,3,4,5,6,7,8] and other tumours such as paragangliomas or suprarenal adenomas [9,10]. Homozygous carriers of *FH* suffer fumarate hydratase deficiency (FHD; OMIM 606812) or fumaric aciduria characterized by facial and brain abnormalities and very serious encephalopathy [11]. The development of these patients is severely affected, with little life expectancy, so it is very important to offer appropriate genetic counselling to heterozygous carriers.

Studies on more than 300 affected families have been published worldwide, but with no data on prevalence [8,12]. Several large series have been published in the USA and Europe, but until now there are no reports of cases of HLRCC in Spain, although the data of some patients have been included in other series [7,8,13,14]. Here, we describe the clinical and genetic characteristics of carriers of *FH* mutations in the largest series of patients diagnosed to date with HLRCC in Spain.

## 2. Materials and Methods

### 2.1. Patients and Data Collection

We included patients with clinical criteria of HLRCC and confirmed family diagnosis by genetic testing on confirmed and obligated carriers between 2009 and 2019 from 11 different centers in Spain. We created a database to collect clinical information from the medical records, such as presence and age of diagnosis of CLMs, ULMs, leiomyosarcoma, RCys and RCCs, hysterectomy and age at surgery, treatment of RCCs, presence and age of diagnosis of other tumours, presence of risk factors for RCCs such as high blood pressure, tobacco use (smoking or passive smoking) and obesity (defined as a body mass index >30 kg/m^2^), and if they did vigorous physical activity, defined as playing sports more than 3 days per week (as a possible trigger of tumour development by increasing metabolic energy demand). RCCs and other cancers were confirmed by histopathology of resected tumours. RCys and adrenal adenomas were diagnosed by imaging procedures such as computed tomography scans, magnetic resonance imaging or ultrasonography. Some RCys were confirmed by histopathology of resected tumours.

### 2.2. Detection of Germline Mutations

DNA from peripheral blood samples was used for *FH* genetic testing. The whole coding sequence and intron-exon boundaries were analysed by polymerase chain reaction amplification and Sanger sequencing for single nucleotide variants and insertion/deletion type of variants. Copy number variations/variants were analysed by multiplex ligation-dependent probe amplification (MLPA) according to the manufacturer’s protocol (SALSA MLPA Probemix P198, MRC-Holland, The Netherlands). Alternatively, next-generation sequencing and further confirmation of findings by Sanger sequencing or MLPA, depending on the variant type, were also performed in a subset of cases. Similarly, relatives at risk were tested using Sanger sequencing or MLPA. The variants were described using the current version of human genomic variant search nomenclature [15], considering LRG_504t1 (NM_000143.3) as the transcript reference sequence. The clinical significance of variants was assessed using the American College of Medical Genetics and Genomics criteria [16]. Over 70 genomic databases were considered through the VarSome [17] data mining tool, including ClinVar [18]. ClinVar provides supporting evidence on the relationships among human genetic variations and phenotypes.

All patients with a genetic diagnosis gave written informed consent for genetic testing according to the Spanish legislation. This study was approved by the Research Ethics Committee of the Hospital General Universitario de Elche on 25 January 2018 (code PI 42/2017).

### 2.3. Statistics

Statistical analyses were performed using R statistical software version 3.6.0 (R Foundation for Statistical Computing). Concerning the descriptive analysis, the qualitative variables are presented as percentages, the continuous quantitative variables are described as the mean and standard deviation (SD) or as the median and the interquartile range (IQR). Categorical variables were compared using chi-squared and Fisher’s exact tests and multivariate logistic regression. Odds ratio (OR) was calculated to estimate de strength of association between variables. Confidence level used was 95% confidence interval (95%CI). Significance was accepted at *p* ≤ 0.05. Cumulative incidence of events was estimate with the cumulative hazard function. Overall survival was estimated by Kaplan-Meier method.

## 3. Results

We identified 197 heterozygous carriers of 27 germline variants in *FH*: 74 index cases and 123 relatives belonging to 74 different families. There were 113 women (57.4%) and 84 men (42.6%), with a mean age of 51.1 years (SD 13.4). All 27 variants were class five or four: 13 missense, five frameshift, four large deletions, three splice-site and two nonsense variants. Twelve (44%) were not previously reported in databases [16,17,19] (Table 1, Table S1).

One hundred and four patients from 31 non-related families were heterozygotic carriers of the pathogenic variant c.1118A > G; p.Asn373Ser: 53 women and 51 men. Twenty-two families (95 individuals) originated from the province of Alicante in the south-east of Spain. Considering the population of Spanish origin in this province, the estimated prevalence is 6.7/100,000 inhabitants.

In looking for genotype-phenotype associations, the pathogenic and probably pathogenic genetic variants were classified into two groups: (a) loss of function, which includes those disruptive variants that would generate truncated proteins or loss of protein expression (nonsense, frameshift, consensus splice site and large deletions) and (b) missense variants, where the predicted functional effect would be milder.

### 3.1. Cutaneous Leiomyomas

**Figure 1:**
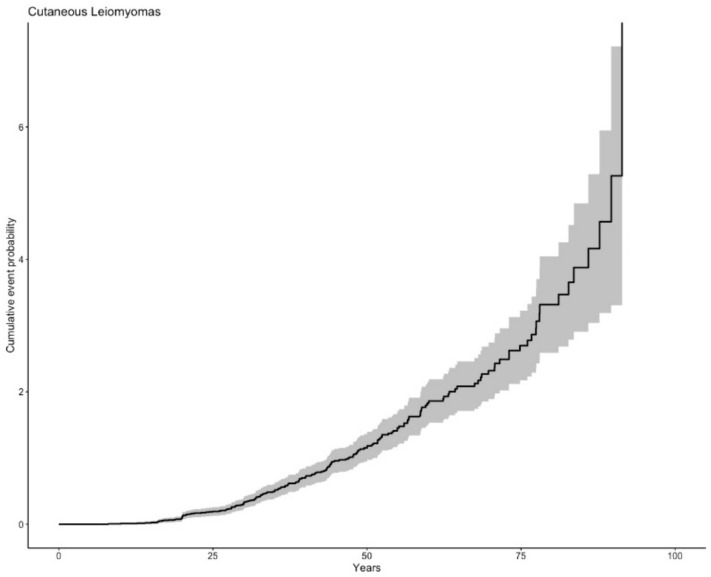
Cumulative incidence of cutaneous leiomyomas.

One hundred and eighteen of 182 patients with dermatologic examination results presented with CLMs (64.8%), 69 women (58.5%) and 49 men (41.5%). The median age of appearance or diagnosis was 36.2 years, (IQR 28.0–48.3) (Figure 1). Two cases (1.7%)—one woman and one man—developed cutaneous leiomyosarcoma at the age of 46 years and 56 years, respectively. The frequency of CLMs was higher in individuals with missense than those with loss-of-function (LoF) variants (68.8% vs. 51.2%; *p* = 0.038; OR 0.47, (95%CI 0.23–0.96)) (Table 2). The cumulative incidence of CLM stratified by variant type did not show significant differences (Figure S1). Univariate analyses and multivariate logistic regression analysis did not reveal differences according to gender, blood pressure status, tobacco use, obesity or use of vigorous physical activity (Tables S2 and S3).

### 3.2. Uterine Leiomyomas

**Figure 2:**
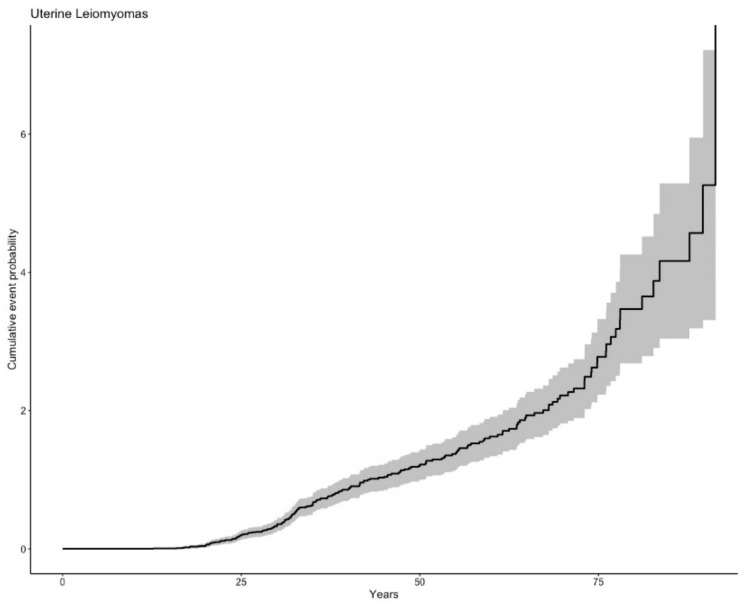
Cumulative incidence of uterine leiomyomas.

Of 103 women with gynaecological examinations, 93 (90.3%) had ULMs, at a median age of 30.7 years (IQR 24.9–34.2) (Figure 2). The frequency of ULMs was higher in individuals with missense than those with LoF variants (96.2% vs. 72%; *p* = 0.001; OR 0.10, (95%CI 0.02–0.43)) (Table 2). The cumulative incidence of ULM stratified by variant type did not show significant differences (Figure S2). The univariate analyses and multivariate logistic regression did not find differences according to blood pressure status, tobacco use, obesity or use of vigorous physical activity (Tables S2 and S3). Hysterectomy was performed in 55.4% of women, at a median age of 34.4 years (IQR 32.0–38.9). In one patient aged 21 years, the pathological diagnosis was leiomyosarcoma (1.3%), and she received pelvic radiotherapy after surgery. A recent pathology review has confirmed the diagnosis of atypical leiomyoma in this case.

### 3.3. Renal Cysts

Of 153 patients with radiological records, 57 (37.3%) presented with RCys, 33 women (58%) and 24 men (42%), at a median age of 53.3 years (IQR 37.2–62.9). One operated patient presented with a haemorrhagic renal cyst with atypia. The frequency was higher in individuals with missense than those with LoF variants (42.6% vs. 21.1%; *p* = 0.017; OR 0.35, (95%CI 0.15–0.85)) (Table 2). The cumulative incidence of RCy stratified by variant type did not show significant differences (Figures S3 and S4). Univariate analysis showed no differences according to gender, blood pressure status or vigorous physical activity, but subjects with tobacco use (*p* = 0.076) and obesity (*p* = 0.058) showed a trend to develop RCys. The multivariate logistic regression did not find differences according to blood pressure status, tobacco use, obesity or use of vigorous physical activity (Tables S2 and S3). A logistic regression model including type of variant and tobacco exposure showed that patients with LoF variants had less risk of developing RCys with tobacco exposure compared with patients with missense variants, but this lacked statistical significance (adjusted OR 2.05, (95%CI 0.96–4.39)) (Table 3).

### 3.4. Renal Cell Cancers

**Figure 3:**
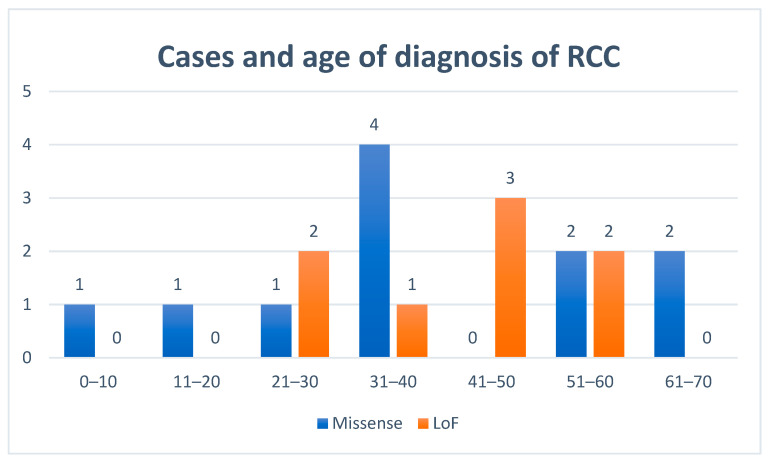
Age at diagnosis of renal cell cancer. RCC, renal cell cancer.

Of 175 patients with confirmed clinical information, 19 presented with RCC (10.9%), 11 men and eight women; fourteen were index cases. Seven of them also presented with RCys. One patient was diagnosed with two synchronous bilateral RCCs. The median age at diagnosis was 37.4 years ((IQR 30.2–53.2), range 10–67) (Figure 3). Ten cases were heterozygote carriers of the pathogenic variant c.1118A > G; p.Asn373Ser, three of them at a very early age (a boy at 10 years, a young man at 20 years and a woman with bilateral RCCs at 24 years). No significant differences were observed according to the type of variant, sex, blood pressure status, tobacco use or obesity in the univariate and multivariate logistic regression model, but those undertaking vigorous physical activity showed a non-significant trend to develop RCCs in the multivariate logistic regression model (*p* = 0.067) (Tables S2 and S3).

**Figure 4:**
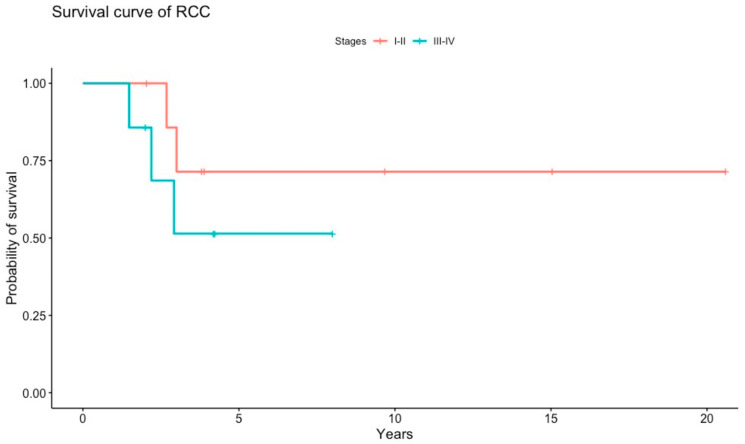
Kaplan-Meier estimates of overall survival of patients with renal cell cancer stages I and II vs. stages III and IV. RCC, renal cell cancer.

Histopathology revealed various patterns of RCCs. Papillary morphology was the most frequent (*n* = 14): 10 cases of type 2 papillary and four papillary type without further subclassification. Four were clear cell carcinoma and two were unclassified. Seven tumours presented with cystic components. One case was diagnosed after genetic testing by imaging screening over an RCy considered previously to be benign. According to the initial presentation, six were stage I, two were stage II, three were stage III, four were stage IV, and in four cases this information was not available. The median overall survival (mOS) was 8.01 years (95%CI 4.0–12.8). For patients with stages I and II, the mOS was not reached, and the mean overall survivals were 15.5 years (95%CI 9.6–21.4). In patients with stages III and IV, the mOS was 2.9 years (95%CI 1.4–4.4) (Figure 4).

**Figure 5:**
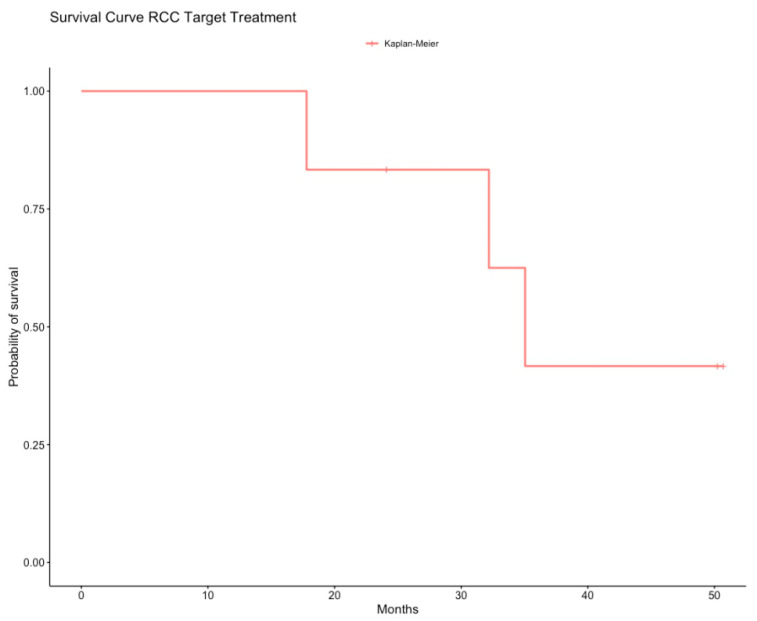
Kaplan-Meier estimates of overall survival of renal cell cancer in patients treated with target treatment (anti-angiogenic and immunotherapy). RCC, renal cell cancer.

Seven patients with metastatic disease received medical treatments (anti-angiogenics with and without immunotherapy) with a mOS of 34.9 months (95%CI: 29.0–40.9) (Figure 5). One patient with a stage I RCC recurred and died at 32.1 months. Of two patients with stage III RCC who recurred, one is alive with complete remission. Two of four patients with stage IV RCC are alive and ongoing treatment at 24 and 50.7 months, respectively. One patient with a stage I RCC had synchronous advanced bladder cancer and died at 35.9 months from diagnosis.

### 3.5. Other Tumours

Twenty-seven of 171 patients with confirmed clinical information (15.8%), 13 women and 14 men, presented with other different tumours, some of them with two. Of 153 patients with radiological records, nine were diagnosed with adrenal adenomas (5.9%), at a median age of 55.1 years (IQR 46.6–59.8). Malignant tumour types were five prostate cancers, three bladder cancers, two colon cancers, two breast cancers, two cutaneous basal cell carcinomas, two cutaneous squamous cell carcinomas, two non-Hodgkin lymphomas, one melanoma, one lung cancer, one pancreatic cancer and one carcinoid tumour.

## 4. Discussion

HLRCC is a very rare form of genodermatosis characterized by the presence of CLMs, ULMs and renal tumours. Despite its high penetrance [2,3,4,5,6,7,8], it is frequently underdiagnosed because the skin lesions can be scarce and patients do not seek medical advice. There are no data on its prevalence, and it is considered a rare disease [12]. We found that in Alicante, a province in the south-east of Spain, this syndrome is frequent probably because of a founder mutation, c.1118A > G; p.Asn373Ser, and we calculated a prevalence of 6.33/100,000 people. This mutation has been described previously in other series with patients of Spanish origin [7,14].

*FH* is located on chromosome 1q42.1, with 1533 nucleotides and 10 exons. It encodes two isoforms of FH, one localized in mitochondria that participates in the TCA cycle catalysing the hydration of fumarate to l-malate, and one in the cytosol that participates in the urea cycle and arginine metabolism [20]. Around 180 pathogenic variants and 82 likely pathogenic types causing HLRCC or FHD have been reported, mainly missense mutations, but there are also frameshift, nonsense, splicing and deletion variants [17,19]. Here we describe 27 pathogenic/likely pathogenic variants, 12 (44%) of them not previously reported in databases [18,21]. Fourteen variants were classified as disruptive pathogenic variants (named LoF), and 13 were missense pathogenic variants.

FH is a homotetramer protein with four active sites of interaction, all of them evolutionarily conserved, formed by amino acid residues 176–193, 228–247 and 359–381. Mutations that affect these regions or the protein structure decrease its enzymatic capacity [22]. Total loss of FH function might be lethal for cells because of its essential role in cellular metabolism. Thus, if the germline mutation generates total loss of function, such as a premature stop codon or large rearrangements, only those somatic second hits with a partial inactivation such as missense or in-frame indel mutations will have a potential oncogenic effect with cells retaining residual enzymatic function. Loss of heterozygosity has been described as the most frequent event for inactivation of the second allele in RCCs and ULMs from patients with HLRCC [2,23]. With these considerations in mind, we hypothesized an association among missense germline pathogenic variants and the HLRCC clinical phenotype. We found significant associations with ULMs, CLMs and RCys, but not with RCCs; however, because of the small number of cases, no firm conclusions can be drawn. Further studies are needed to evaluate potential differences in penetrance related to the type of genetic variant in *FH*.

Several mechanisms may be involved in the development of these tumour types. A deficit of FH leads to blockage of the TCA, with the accumulation of fumarate and succinate, and a decrease in oxidative phosphorylation. To compensate for this, there is a metabolic shift to aerobic glycolysis for energy production that increases lactate and reactive oxygen species (ROS) levels [24]. ROS and high levels of fumarate inhibit the degradation of hypoxia-inducible factor alpha (HIFα) by O2-dependent prolyl hydroxylases leading to a situation of pseudohypoxia [25,26]. The accumulation of fumarate and succinate produces succination in cysteine residues of proteins, S-(2-succinyl) cysteine (2SC), such as the transcription factor Kelch-like ECH-associated protein 1 (KEAP1), resulting in upregulation of genes involved in the antioxidant response such as those encoding nuclear erythroid 2-like 2 transcription factor (NRF2) that leads to cell proliferation [27,28]. In addition, elevated levels of fumarate cause epigenetic suppression of a family of anti-metastatic microRNAs such as MIR200, leading to epithelial-to-mesenchymal transition, a phenotypic switch that promotes tumour formation and metastasis [29].

CLMs are benign tumours that arise from the arrector pili musculature. These muscles participate in thermoregulation and in response to strong emotions, but in humans their action is considered a vestigial reflex. In our patients, CLMs were present in 64.8%, similar to other series in the literature (48.6–84%) [6,7,8,30]. Some patients were previously misdiagnosed as having post-acne scarring or other chronic cutaneous lesions as described by several authors [3,4,6,7]. Many of the relatives of our patients were diagnosed by dermatologists after positive genetic testing because they did not notice the lesions. Patients with missense variants presented with a higher frequency of CLMs than those with LoF variants, which have not been described hitherto in other series. However, no significant differences were observed in the cumulative incidence when stratified by variant type. Two patients developed cutaneous leiomyosarcomas (1.7%), although few such cases have been described [4,31].

Almost all women (77–100%) with LHRCC develop numerous and large ULMs at a younger age than sporadic ones. In our series, 90.3% of women were diagnosed with leiomyomas at similar ages to those described previously [2,3,4,5,6,7,8,9,32]. We observed a higher frequency in women with missense compared with LoF variants, and this has also not been described before. Variant types did not show differences in the cumulative incidence. In some cases, myomectomy was the surgical treatment, but hysterectomy was performed in 55.4% at a mean age of 34.4 years as the best treatment for their symptoms. Transformation to leiomyosarcomas have been described, but new diagnostic guidelines actually classify these tumours as atypical leiomyomas [4,9,23]. Sporadic leiomyomas develop during the reproductive period and regress after menopause [33] and show increased expression of oestrogen and progesterone receptors and hypoxia-induced angiogenic factors (HIF) compared with normal myometrium [34]. The ULMs of patients with HLRCC are more vascularized and show more overexpression of HIF1α and vascular endothelial growth factor (VEGF) than do sporadic leiomyomas [35,36], and this should be considered by gynaecologists to prevent haemorrhagic complications at the time of surgery.

An association between RCys and RCCs has been postulated [37]. Patients with inherited RCC syndromes as Von Hippel Lindau syndrome, tuberous sclerosis complex, and HLRCC also develop RCys. In the general population, the prevalence of RCys is 4.6–8.2% compared with 36% in patients with HLRCC, presenting at a younger age [9]. A study in *FH*-deficient mice demonstrated that RCys formation is mediated by NERF2 but is hypoxia-inducible factor (HIF)-independent [38]. Benign cyst models in *FH*-deficient cells show 2SC staining, suggesting that such cysts are the precursor lesions of RCCs [39]. The use of the Bosniak classification for RCys does not seem to be adequate in these patients because of the higher risk of malignancy. In our series, 37.3% of the patients had RCys, and 7/19 of RCCs had confirmed cystic components. Patients with missense variants presented with RCys more frequently than those with LoF variants. This difference has not been described in other series. No differences in the cumulative incidence were detected by variant type.

RCCs are not a single disease but several different diseases with different histological patterns and prognoses, in which HIFs are the main cornerstones in pathogenesis [40,41]. RCCs must be considered metabolic diseases. In VHL syndrome and sporadic RCCs, germline and somatic mutations in the *VHL* gene lead to upregulation of proangiogenic HIF through the VHL tumour suppressor pathway. In both HLRCC and hereditary paraganglioma-pheochromocytoma syndrome (caused by mutations in the succinate dehydrogenase complex, *SDH*, genes), there is overexpression of HIF in tumour tissues. Both syndromes affect the TCA cycle, and in both there is an inhibition of prolyl hydroxylases and consequent HIF stabilization by accumulated fumarate or succinate. However, there must be other mechanisms involved to explain the different clinical manifestations [42]. The published risk of developing RCCs in patients with HLRCC is 15–34%, at a mean of 44 years of age; more frequent in men; usually unilateral and most often diagnosed at advanced stages with extremely aggressive evolution [2,3,4,5,6,7,8,9,43,44,45,46]. In our series, the rate was lower at 10.9%. This low rate might be more realistic than in other published series selected by the diagnosis of RCCs. The median age in our patients is lower (37.4 years), including four cases younger than 25 years old. Tobacco smoking, hypertension and obesity are established risk factors for RCC, which can be modestly reduced by physical activity. RCC incidence is two-fold higher in men than in women [47]. No significant differences were found for these factors.

For many years, type 2 papillary RCC has been associated specifically with HLRCC, but different communications have described other types as tubulocystic carcinoma, collecting duct renal carcinoma, oncocytic or even clear cell carcinoma (in our series four cases). In the 2016 WHO Classification of Tumours of the Urinary System, this was proposed as a new entity, named HLRCC-associated RCC (code 8311/3) [48], with variable pathological features but with a specific papillary architecture with abundant eosinophilic cytoplasm, large nuclei, very prominent nucleoli, perinucleolar halos and usually Fuhrman grade three to four. This emphasizes the importance of looking for subcellular characteristics in histopathology and the use of immunohistochemical techniques for detecting 2SC residues or FH to avoid misdiagnosis. Unlike other published series, in our study the majority of patients with RCCs diagnosed as stages I–II had similar survival to sporadic cases. The mean of survival was longer than that published in another series (15.5 vs. 7.3 years) [30].

There are no data published of results of specific treatment phase III clinical trials for this syndrome, so the treatment must be based on antiangiogenic and immunotherapy approaches as in other subtypes of RCCs. Ravaud et al. [49] published a phase II study on patients with advanced or metastatic papillary type 1 and 2 RCCs treated with sunitinib as a first-line therapy. The mOS was 17.8 months (95% CI 5.7–26.1) and 12.4 (95% CI 8.2–14.3) months for types 1 and 2, respectively. Srinivasan et al. [50] presented a phase II study in the American Society of Clinical Oncology meeting of 2020 with bevacizumab and erlotinib for patients with metastatic HLRCC-associated RCCs and metastatic sporadic papillary RCCs. The overall response rate was 51% (95% CI 40–61) in all patients, and 64% (95% CI 49–77) in the HLRCC cohort; the median progression-free survival was 14.2 months (95% CI, 11.4–18.6) in all patients, and 21.1 months (95% CI, 15.6–26.6) in the HLRCC cohort. A phase I/II trial using vandetanib and metformin in patients with advanced RCCs associated with HLRCC or a *SDH* mutation, and sporadic/non-HLRCC papillary type RCCs is ongoing [51]. Immuno-oncology treatment has shown promising results in patients with papillary RCCs. Patients treated with a combination of savolitinib and durvalumab had a mOS of 12.3 months (95%CI 5.8–21.3) [52]. In our series, patients with metastatic RCCs treated with antiangiogenic therapy with or without immunotherapy had a mOS of 35 months.

Other tumours have been associated with this syndrome, such as adrenocortical adenomas and carcinomas, Wilm’s tumour, Leydig cell tumours, gastrointestinal stromal tumours, ovarian cystadenomas, pheochromocytomas and paragangliomas [8,10,53]. The risk of pheochromocytomas and paragangliomas must be taken into account in those patients with a diagnosis of adrenal tumours made by imaging procedures. To date, we have not diagnosed any cases of pheochromocytomas or paragangliomas in our series. We observed nine (5.9%) patients with adrenal adenomas, at a median age of 55.1 years (range 46.6–59.8). Radiological studies report a frequency of this tumour of around 4.5% in general populations [54]. We did not find any increased incidences of other cancer types.

However, our study had limitations because of the lack of complete clinical information for some patients. Nevertheless, this is one of the largest series ever published of patients with HLRCC. Our objective is to continue a prospective phase to evaluate phenotypes and the results of screening RCCs in at-risk patients.

In the Second Symposium on Hereditary Leiomyomatosis and Renal Cancer held in Paris in 2013, the consensus recommended—preferably—yearly renal MRI scans beginning at 8–10 years of age, although the risk is low up to age 20 [46,55]. Given our results, we support this, especially in patients with the mutation c.1118A>G who have RCCs at 10, 20 and 24 years of age. Screening with ultrasonography alone is not recommended because the lesions may be isoechoic and not detected. Bosniak criteria [56] to classify RCys are not reliable in these patients.

There are many questions about this syndrome. Why are the clinical manifestations so characteristic? Why are other smooth muscle tissues and localizations not affected? Is it possible to stop neoplastic transformation by targeting the altered metabolic route? We believe that research should focus on the embryonic development of these tissues. For instance, nephronectin, an extracellular matrix protein, binds α8β1 integrin in early kidney development and in arrector pili muscle progenitors [57,58]. Animal models have been designed to investigate some of the predisposition to RCC syndromes, such as Eker rats with pathogenic variants in the *TSC2* gene, German Shepherd dogs with pathogenic variants in the *BHD* gene and *FH*-deficient mice [59,60]. All these syndromes show some similarities in associated tumours (RCys and RCCs, uterine myomas and cutaneous lesions).

## 5. Conclusions

This is the first report of a large series of patients with HLRCC syndrome from Spain. The clinical manifestations were similar to those described in other series, but we have found phenotypic differences in patients with missense mutations who have a higher frequency of CLMs, ULMs and RCys. Established risk factors for RCC have been analysed. The frequency of RCCs was lower than those reported in the previously published series. RCys must be closely monitored as potential precursors of RCCs, with CT or preferably MRI as the optimal imaging techniques for the early detection of RCCs. Papillary type 2 is the most frequent in terms of histopathology, but other histological patterns do not exclude this syndrome. In the province of Alicante, there is a higher prevalence. Alerting health professionals such as family doctors, dermatologists, gynaecologists, radiologists, pathologists or urologists might help in early and correct diagnoses and in offering genetic counselling to heterozygous carriers about the risk of RCCs, leiomyomas and their complications, as well as the risk of FHD in offspring.

## Figures and Tables

**Table 1:** Genotype and phenotype characteristics of HLRCC families.

Variant	Class	Variant Type	No. Families/ No. Individual (Male; Female)	CLM *	ULM *	RCy *	RCC *	Origin
				*No. affected patients/Total no*				
**Del *FH***	5	LoF	2/6 (4; 2)	2/6	1/2	2/6	0/6	Spain
Del exon 2	5	LoF	1/1 (0; 1)	1/1	0/1	0/1	1/1	Spain
c.139C > T	5	LoF	1/1 (0; 1)	1/1	1/1	?	0/1	Spain
c.267 + 1_267 + 10del	5	LoF	1/1 (0; 1)	1/1	0/1	0/1	0/1	Spain
c.301C > T	5	LoF	1/1 (0; 1)	1/1	1/1	1/1	0/1	Spain
c.349G > C	4	Missense	2/3 (1; 2)	2/2	2/2	?	0/3	Spain
c.395delT	5	LoF	1/16 (5; 11)	1/15	6/10	0/14	2/16	Spain
c.553delC	5	LoF	1/1 (0; 1)	1/1	1/1	?	?	Spain
c.555 + 1G > A	5	LoF	1/1 (0; 1)	1/1	1/1	1/1	0/1	Spain
c.563A > G	4	Missense	1/2 (1; 1)	1/2	0/1	0/1	0/1	Spain
c.575C > T	4	Missense	4/11 (4; 7)	11/11	7/7	5/11	0/11	Spain
c.697C > T	4	Missense	5/10 (5; 5)	5/9	4/4	3/6	0/6	Spain
c.698G > A	5	Missense	4/7 (2; 5)	4/6	4/4	1/1	1/3	Spain
c.703C > T	4	Missense	1/1 (0; 1)	?	?	?	0/1	Spain
c.845G > T	4	Missense	3/6 (2; 4)	5/5	3/4	3/4	1/5	Spain
c.893del	5	LoF	1/2 (0; 2)	2/2	1/1	0/1	0/1	Spain
c.905-2A > G	5	LoF	1/1 (1; 0)	1/1	-	1/1	0/1	Spain
c.965T > G	4	Missense	1/1 (0; 1)	1/1	1/1	0/1	0/1	Spain
c.974delG	5	LoF	1/3 (1; 2)	1/1	?	0/1	1/1	Spain
Del exon 8	5	LoF	3/6 (1; 5)	4/5	4/4	3/6	1/6	Spain
c.1112A > G	4	Missense	1/1 (1; 0)	1/1	-	0/1	0/1	Perú
c.1118A > G	5	Missense	31/104 (51; 53)	64/99	50/51	36/85	10/101	Spain
c.1126delC	5	LoF	1/6 (4; 2)	5/6	2/2	0/6	0/6	Spain
c.1189G > A	4	Missense	2/2 (0; 2)	1/2	2/2	0/2	0/2	Colombia
c.1217A > C	4	Missense	1/1 (1; 0)	1/1	-	0/1	0/1	Cuba
c.1240A > G	4	Missense	1/1 (0; 1)	0/1	1/1	1/1	1/1	Belarus
Del exon 9	5	LoF	1/1 (0; 1)	?	1/1	?	1/1	Spain
			74/197 (84; 113)	118/182 (64.8%)	93/103 (90.3%)	57/153 (37.3%)	19/175 (10.9%)	
Loss of Function			17/47 (16; 31)	21/41 (51.2%)	18/25 (72.0%)	8/38 (24.8%)	6/42 (14.3%)	
Missense			57/150 (68; 82)	97/141 (68.8%)	75/78 (96.1%)	49/115 (42.6%)	13/133 (9.8%)	

HLRCC, Hereditary leiomyomatosis and renal cell cancer; CLM, cutaneous leiomyomas; ULM, uterine leiomyomas; RCy, renal cysts; RCC, renal cell cancer; LoF, Loss of Function; * Confirmed clinical information; ?, no information.

**Table 2:** Phenotype/Genotype associations.

Clinical Manifestations	Missense No. Affected/Total (%)	LoF No. Affected/Total (%)	OR (95%CI)	*p*-Value *
CLM	97/141 (68.8)	21/41 (51.2)	0.47 (0.23–0.96)	0.038
ULM	75/78 (96.1)	18/25 (72.0)	0.10 (0.02–0.43)	0.001
RCy	49/115 (42.6)	8/38 (21.0)	0.35 (0.15–0.85)	0.017
RCC	13/133 (9.7)	6/42 (14.2)	1.53 (0,55–4,34)	0.412

CLM, cutaneous leiomyomas; ULM, uterine leiomyomas; RCys, renal cysts; RCC, renal cell cancer; LoF, loss of function; OR, odds ratio; C: confidence interval; * X^2^ test.

**Table 3:** Multivariate logistic regression model for Renal Cyst, variant type and tobacco variables.

Variable	RCy	
	OR (95%CI)	*p*-value *
Variant type		
Missense	Ref.	
Lof	0.23 (0.08–0.65)	0.005
Tobacco		
No	Ref.	
Yes	2.05 (0.96–4.39)	0.065

RCy: Renal Cyst; LoF: Loss of Function; OR: Odds Ratio; * Wald’s test.

## Supplementary Materials

The following are available online at https://www.mdpi.com/2072-6694/12/11/3277/s1, Figure S1: Cumulative incidence of cutaneous leiomyomas by variant type. Light-colored areas represent confidence intervals, Figure S2: Cumulative incidence of uterine leiomyomas by variant type. Light-colored areas represent confidence intervals, Figure S3: Cumulative incidence of renal cysts, Figure S4: Cumulative incidence of renal cysts by variant type. Light-colored areas represent confidence intervals, Table S1: *FH* germline variants classifications and phenotype characteristics of HLRCC families, Table S2: Univariate analysis for cutaneous leiomyomas, uterine leiomyomas, renal cysts, renal cell cancer and risk factors, Table S3: Multivariate logistic regression model for cutaneous leiomyomas, uterine leiomyomas, renal cysts, renal cell cancer and risk factors.
