# Supplementary material for: Hereditary Leiomyomatosis and Renal Cell Cancer Syndrome in Spain: Clinical and Genetic Characterization
Source: Cancers (Basel). 2020 Nov 5;12(11):3277. doi: 10.3390/cancers12113277 (PMC7694543; doi:10.3390/cancers12113277)
Supplement: Supplementary file 1 [file cancers-12-03277-s001.zip › cancers-970039-supple-xml/cancers-970039-suppl-xml.pdf]

Supplementary Materials: Hereditary Leiomyomatosis and Renal Cell Cancer Syndrome in Spain: Clinical and Genetic Characterization

A. Beatriz Sánchez-Heras, Adela Castillejo, Juan D. García-Díaz, Mercedes Robledo, Alexandre Teulé, Rosario Sánchez, Ángel Zúñiga, Enrique Lastra, Mercedes Durán, Gemma Llort, Carmen Yagüe, Teresa Ramon y Cajal, Consol López San Martin, Adrià López-Fernández, Judith Balmaña, Luis Robles, José M. Mesa-Latorre, Isabel Chirivella, María Fonfria, Raquel Perea Ibañez, M. Isabel Castillejo, Inés Escandell, Luis Gomez, Pere Berbel and Jose Luis Soto

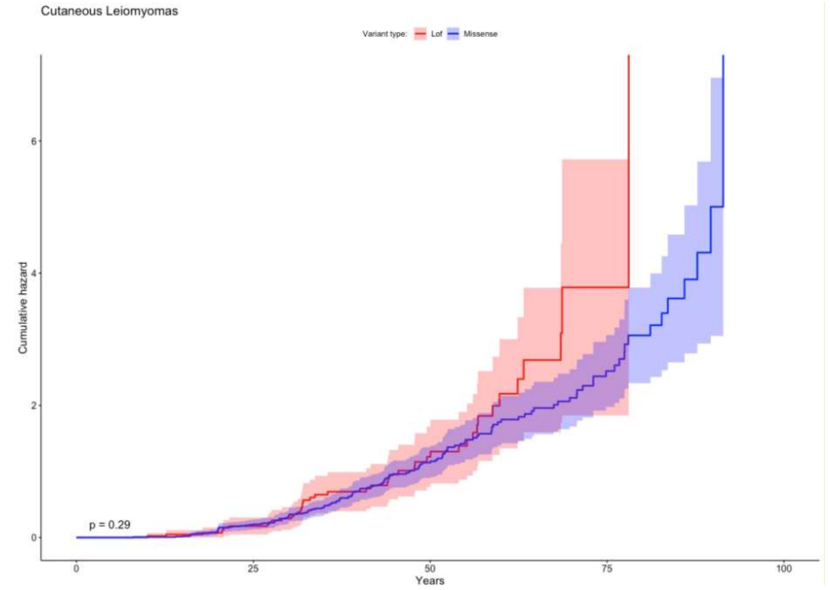

Figure S1. Cumulative incidence of cutaneous leiomyomas by variant type. Light-colored areas represent confidence intervals.

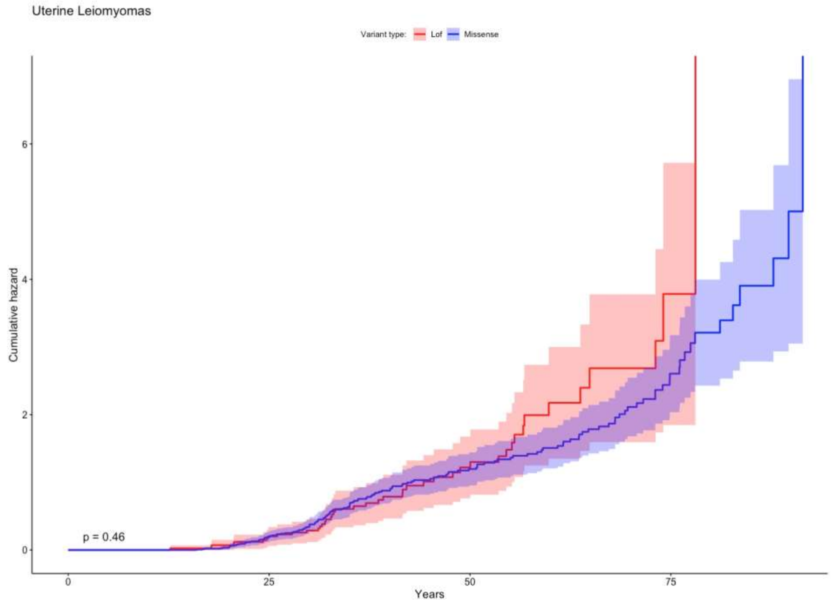

Figure S2. Cumulative incidence of uterine leiomyomas by variant type. Light-colored areas represent confidence intervals.

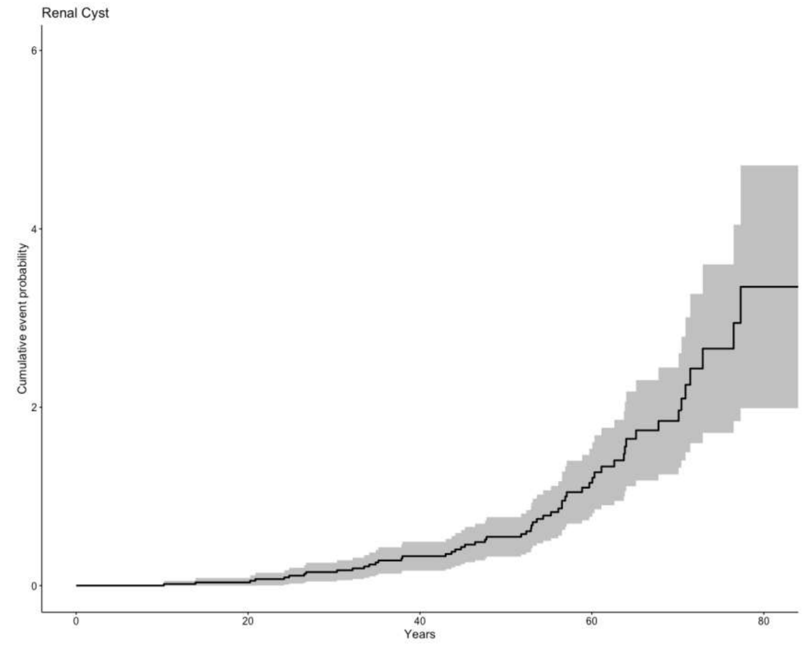

Figure S3. Cumulative incidence of renal cysts.

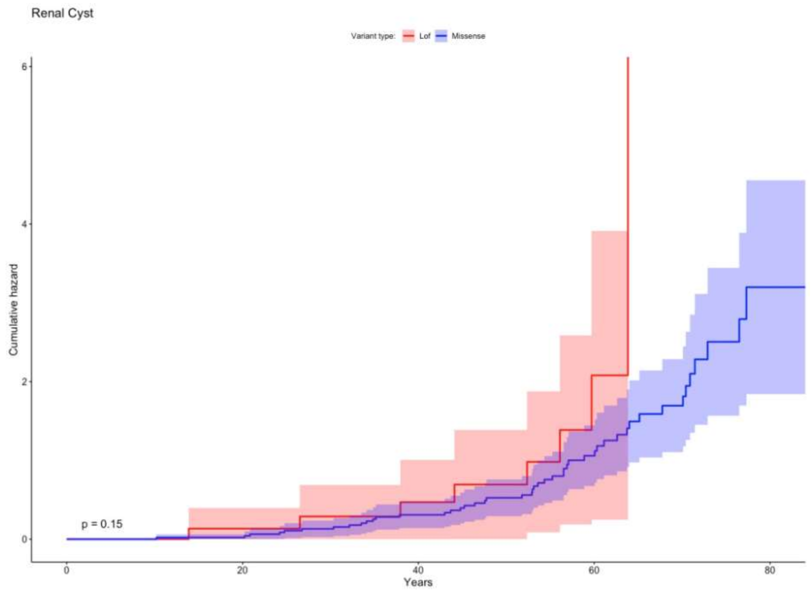

Figure S4. Cumulative incidence of renal cysts by variant type. Light-colored areas represent confidence intervals.

Table S2. Univariate analysis for Cutaneous Leiomyomas, Uterine Leiomyomas, Renal Cysts, Renal Cell Cancer and risk factors.

**Clinical Manifestations**	**CLM**		**ULM**		**RCy**		**RCC**	
**Variable**	**N af /T (%)**	***p*-Value**	**N af T/ (%)**	***p*-Value**	**N af / T (%)**	***p*-Value**	**N af / T (%)**	***p*-Value**
Variant type								
Missense	97/141 (68.8)	0.038*	75/78 (96.1)	0.001*	49/115 (42.6)	0.017*	13/133 (9.8)	0.412*
Lof	21/41 (51.2)		18/25 (72)		8/38 (21))		6/42 (14.3)	
Gender		0.272*		-		0.566*		0.236*

Male	49/81 (60.5)		–		24/69 (34.8)		11/79 (14)	
Female	69/101 (68.3)		-		33/84 (39.3)		8/96 (8.3)	
Obesity								
No	92/149 (61.7)	0.669*	71/80 (88.7)	0.696*	47/136 (34.5)	0.058*	14/148 (9.5)	1†
Yes	10/14 (71.4)		8/8 (100)		8/12 (66.6)		1/13 (7.7))	
HBP								
No	84/132 (63.6)	0.848*	67/76 (88.1)	0.548*	40/119 (33.6)	0.156*	11/129 (8.5)	1†
Yes	19/29 (65.5)		10/10 (100)		13/27 (48.1)		3/30 (10)	
Phy.activity								
No	82/136 (60.3)	0.378*	67/75 (89.3)	1†	44/123 (35.7)	0.813†	9/134 (6.7)	0.126†
Yes	10/13 (76.9))		5/5 (100)		3/11 (27.3)		3/13 (23.1)	
Tobacco								
No	59/102 (57.8)	0.196*	55/62 (88.7)	1*	29/93 (31.2)	0.076*	9/102 (8.8)	0.510*
Yes	35/51 (68.6)		18/20 (90)		21/45 (46.6)		6/49 (12.2)	

CLM: cutaneous leiomyomas; ULM: uterine leiomyomas; RCy: renal cyst; RCC: renal cell carcinoma; N af, no. affected; T, total; LoF: Loss of Function; HBP: high blood pressure; Phy.activity: vigorous physical activity; *X^2 test; †Fisher's exact test.

Table S3. Multivariate logistic regression model for Cutaneous Leiomyomas, Uterine Leiomyomas, Renal Cysts, Renal Cell Cancer and risk factors.

**Clinical Manifestations**	**CLM**		**ULM**		**RCy**		**RCC**	
**Variable**	**OR (95% CI)**	***p*-Value***	**OR (95% CI)**	***p*-Value***	**OR (95% CI)**	***p*-Value***	**OR (95% CI)**	***p*-Value***
Variant type								
Missense	Ref.	-	Ref.	-	Ref.	-	Ref.	-
Lof	0,27 (0,11-0,64)	0,003	0,07 (0,01-0,43)	0,004	0,23 (0,07-0,64)	0,013	0,98 (0,23-4,12)	0,978
Obesity								
No	Ref.	-	Ref.	-	Ref.	-	Ref.	-
Yes	1,18 (0,32-4,29)	0,801	Inf (0, inf)	0,996	2,51 (0,66-9,56)	0,177	1,16 (0,12-11,45)	0,896
HBP								
No	Ref.	-	Ref.	-	Ref.	-	Ref.	-
Yes	1,11 (0,43-2,86)	0,836	Inf (0, inf)	0,996	1,44 (0,54-3,86)	0,468	0,97 (0,18-5,19)	0,968
Phy.activity								
No	Ref.	-	Ref.	-	Ref.	-	Ref.	-
Yes	2,81 (0,67-11,71)	0,156	Inf (0, inf)	0,997	0,83 (0,19-3,59)	0,804	4,21 (0,9-19,55)	0,067
Tobacco								
No	Ref.	-	Ref.	-	Ref.	-	Ref.	-

Yes	1,55 (0,71-3,40)	0,272	1,43 (0,13-15,96)	0,77	2,43 (1,07-5,509	0,033	0,93 (0,25-3,5)	0,916

CLM: cutaneous leiomyomas; ULM: uterine leiomyomas; RCy: renal cyst; RCC: renal cell carcinoma; LoF: Loss of Function; OR: Odd Ratio; HBP: high blood pressure; Phy.activity: vigorous physical activity; * Wald's test.

© 2020 by the authors. Licensee MDPI, Basel, Switzerland. This article is an open access article distributed under the terms and conditions of the Creative Commons Attribution (CC BY) license (http://creativecommons.org/licenses/by/4.0/).
